# Mediating effect of coping style between empathy and burnout among Chinese nurses working in medical and surgical wards

**DOI:** 10.1002/nop2.584

**Published:** 2020-08-04

**Authors:** Li Cheng, Jiao Yang, Mengyuan Li, Wenru Wang

**Affiliations:** ^1^ School of Nursing Hubei University of Medicine Shiyan China; ^2^ School of Nursing Hubei University of Medicine Shiyan China; ^3^ Institute of Systems Science National University of Singapore Singapore; ^4^ Alice Lee Center for Nursing Studies Yong Loo Lin School of Medicine National University of Singapore Singapore

**Keywords:** burnout, Chinese nurses, coping, empathy, nursing

## Abstract

**Aim:**

This study aimed to investigate burnout, coping style and empathy among Chinese nurses working in medical and surgical wards and to examine the mediating effect of coping style between empathy and burnout among this group of nurses.

**Design:**

A cross‐sectional questionnaire survey.

**Method:**

The study recruited a convenience sample of 363 nurses from three university‐affiliated tertiary hospitals in Shiyan City, Hubei, China. A set of self‐administered questionnaires was used to measure the variables of burnout, coping style and empathy. Structural equation modelling was performed using AMOS 20.0.

**Results:**

Nurse burnout was prevalent among Chinese nurses in medical and surgical wards, and coping style and empathy were significantly associated with burnout. Positive coping strategies and high levels of empathy could reduce burnout in nurses. Coping strategies were found to play a partial mediating role between empathy and burnout among this group of nurses.

## 1 INTRODUCTION

Nurses are the major providers of health care to the community. However, the nature of nursing work in the 21st century can provide challenges to their psychological well‐being. Countries face a rising nursing workforce shortage, which can be attributed to both recruitment and retention issues (Drury, Francis, & Chapman, 2008). Nonetheless, according to the National Health and Family Planning Commission of the People's Republic of China, the country has an increasing number of Registered Nurses (RNs), from 3.24 million in 2015–approximately 4.45 million in 2020 (National Health Commission, 2016).

## 2 BACKGROUND

Across China, hospitals have reported a high turnover rate for nurses. In tertiary hospitals, about 50% of the nurses have expressed their intention to quit (Zhang, Liu, Yang, & Liu, 2015). The demanding nature of nursing work exposes nurses to both acute stress and chronic stress, which can lead to burnout. Nursing burnout is a strong predictor of work‐related intention for turnover (Leiter & Maslach, 2009). Moreover, job burnout affects individuals, organizations and patient outcomes if coping strategies are not in place (Lee, Kuo, Chien, & Wang, 2016). Nurse burnout negatively affects the psychological and physical health of nurses (Meeusen, Van Dam, Brown‐Mahoney, Van Zundert, & Knape, 2010) and critically influences the ability of health care organizations to operate smoothly and sustainably (Maslach, 1982). It is also associated with the negative impact on patient quality care; in other words, patients’ safety can be compromised (Nantsupawat, Nantsupawat, Kunaviktikul, Turale, & Poghosyan, 2016).

According to Maslach (1982, p‐80), burnout refers to “*burn[ing] one's self out*” and is a metaphor to describe people experiencing emotional exhaustion like the draining of a battery. Among individuals who work with people, burnout syndrome and chronic stress reactions consist of emotional exhaustion, depersonalization and reduced personal accomplishment (Maslach, 1982).

Although much is known about the influence of coping styles on nurses’ burnout, most of the studies have explored the relation between empathy and burnout, with few studies investigating the relations among coping style, empathy and burnout in the population of medical and surgical nurses. Moreover, it is unclear whether the relation between empathy and burnout is mediated by coping style. Furthermore, most of the previous studies targeted on the nurses who worked at critical or special wards, for example intensive care unit, emergency department and psychiatry ward. The present study is therefore designed to target on medical and surgical nurses and aims to address the following research questions. (1) What is the status of burnout, coping style and empathy among medical and surgical nurses in China? and (2) What is the mediating effect of coping style between empathy and burnout among the nurses in China?

**Figure 1:**
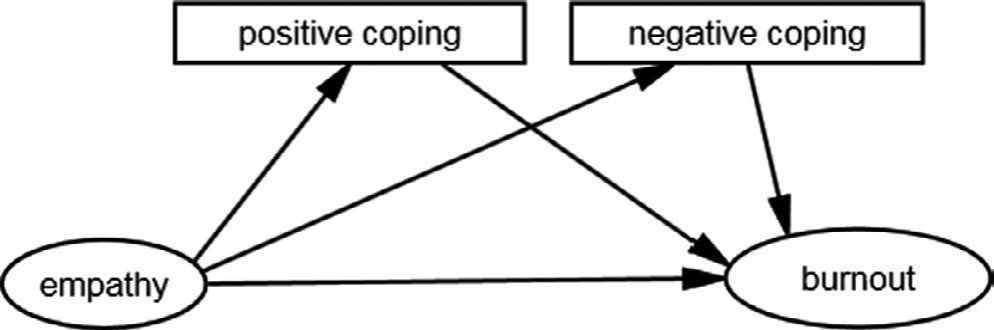
Hypothesized model

In this study, a hypothesized model was developed according to our literature review (Figure 1). In this model, we proposed the relationships among empathy, coping style and burnout among medical and surgical nurses in China. Nurses’ empathy level and coping style are significantly associated with their burnout. We hypothesized that: (1) empathy has significantly negative influence on burnout; (2) positive coping style has significantly negative influence on burnout whereas negative coping style has significantly positive influence on burnout; and (3) coping style is a mediator between burnout and empathy.

## 3 METHODS

### 3.1 Study design and settings

This study adopted a cross‐sectional, descriptive correlational design using self‐reported questionnaires. The study was conducted at three tertiary hospitals in Shiyan, Hubei Province, China.

### 3.2 Study participants

A convenience sample was used. Nurses who met the following inclusion criteria were invited to participate in this study: (1) full‐time RNs working in China; (2) aged 18 years or older; (3) work in a general medical or general surgical ward; and (4) able to read and understand Chinese. We excluded those nurses who worked part‐time and were on leave of absence during the data collection period. To perform a *SEM* analysis, Fan (1999) recommended a sample of at least 200 to ensure the statistical relevance, whereas Stevens (2009) reported that the rule of thumb is to include at least 15 cases per measured variable or empirical indicator. Our study included two latent constructs with eight empirical indicators. Hence, a minimum of 300 participants would be needed in the study; 363 participants were finally included according to the above‐mentioned rules.

### 3.3 Instruments

The questionnaire consisted of items on the socio‐demographic data of the participants and three validated assessment tools, namely Maslach Burnout Inventory‐General Survey (MBI‐GS), Scale of Empathy Competencies of Clinical Nurses (SECCN) and Coping Style Scale (CSS). The socio‐demographic data included gender, age, sibling status, marital status, children, educational level, work unit, number of years in nursing, academic level, job title and employment type.

#### 3.3.1 MBI‐GS

The original MBI‐GS was developed by Maslach to measure job burnout. The English version of MBI‐GS has been translated and validated for Chinese nurses (^Zhu, Lou, & Wang,^ 2007 ^) and^ widely used in assessing the burnout of nurses in China (Huang, Dai, Zhang, Cheng, & Fu, 2013; Liao, Wei, & Hu, 2008; Liu, Li, & Wang, 2012; Luo, Zhang, & Gu, 2013; Tang, Gao, & Zhong, 2017; Wang, Zhu, Yang, & Lou, 2012). The Chinese version of MBI‐GS consists of 15 items grouped under three subscales: emotional exhaustion (5 items), depersonalization (4 items) and personal accomplishment (6 items). Items are rated using a seven‐point Likert scale (0 = never and 6 = everyday), with higher scores indicating higher levels of burnout. The overall score of MBI‐GS is calculated by weighing coefficients and scores of the original response dimensions (0.40 * emotional exhaustion + 0.30 * depersonalization + 0.30 x (6 ‐ personal accomplishment). The level of burnout experienced by nurses was determined by the following cut‐off scores: 0–1.49 indicated no burnout, 1.50–3.49 indicated some burnout and 3.50–6 indicated severe burnout (Kalimo, Pahkin, Mutanen, & Toppinen‐Tanner, 2003). The Chinese version of the MBI‐GS has been demonstrated to have good reliability and validity. The internal consistency of MBI‐GS is acceptable, with Cronbach's alpha ranging from 0.672–0.872 for the subclasses (*p* < .01). The test–retest reliability is also satisfactory, with an intraclass correlation coefficient (ICC) of 0.713 (*p* < .01) (Zhu et al., 2007).

#### 3.3.2 SECCN

In our study, nurses’ level of empathy was measured using the SECCN. The SECCN developed by Wang in 2009 consists of 28 items grouped under three subscales and rated using a five‐point Likert scale ranging from 1 (strongly disagree)–5 (strongly agree). The three subscales are “nurses’ cognition of patients’ psychology” (15 items), “nurses’ experience of patients’ emotion” (5 items) and “nurses’ behaviour in assisting patients” (8 items) (Wang, 2009). The scale has good reliability, with Cronbach's alpha value of 0.86 for the overall scale and 0.71–0.86 for the three subscales (Wang, 2009). The test–retest reliability of the subscales is satisfactory, with ICC of 0.75–0.84.

#### 3.3.3 CSS

We assessed the participants’ coping style using CSS. It is a 20‐item instrument developed by ^Xie (^ 1998 ^)^ using a sample of 846 Chinese urban residents. CSS consists of two subscales: “positive coping style” (12 items) and “negative coping style” (8 items). The scale is rated using a four‐point scale from ‘never’ (0)–‘often’ (3), with higher scores indicating higher levels of coping. It has good reliability, with Cronbach's alpha of 0.78 and 0.90 for the two subscales of positive and negative coping style, respectively. Its test–retest reliability is satisfactory, with ICC of 0.90 for the CSS total and 0.78–0.90 for the subscales (Xie, 1998).

### 3.4 Data collection procedure

The data were collected from January–April 2017. After obtaining the approval from the Research Ethics Committee of Hubei University of Medicine, we informed the nursing directors of the study hospitals regarding the study and sought their permission to conduct the study. We personally distributed the self‐reported questionnaires to the nurses who met the study criteria. The participants were asked to return the completed questionnaire in a sealed envelope to the researchers. The returned completed questionnaire was considered as the participant's informed consent.

### 3.5 Data analysis

Data were analysed using SPSS 20.0 and AMOS 20.0. We used descriptive statistics to analyse the demographic data and the mean and standard deviations of the outcome measures. Pearson correlation coefficient was used to test the correlations among nurses’ burnout, coping style and empathy. In addition, we performed *SEM* analysis to test the hypothesized model. The coping methods were positive and negative coping styles.

### 3.6 Ethical consideration

Institutional review board approval was obtained before the study. Prior to the survey, the researchers contacted the nursing departments of the three hospitals and consent was obtained from their directors. The participants were informed that participation in the study was voluntary and that they could withdraw at any time. Confidentiality and anonymity of data were maintained throughout the study.

## 4 RESULTS

### 4.1 Socio‐demographic characteristics

Among the 380 nurses who received the questionnaires, 372 completed and returned the questionnaires. Of the returned questionnaires, 363 were valid and included in the final data analysis, giving a valid response rate of 95.53%. The socio‐demographic characteristics of the nurses are presented in Table 1. The mean age of the 363 nurses was 29.33 (*SD* 6.16) years, and the average duration of working experience was 7.68 (*SD* 6.96) years.

**Table 1:** Social‐demographic characteristics of the participants (*N* = 363)

Variable	Category	*n*	%	Variable	Category	*n*	%
Gender				work unit			
	Male	8	2.20		medical unit	181	49.86
	Female	355	97.80		surgical unit	182	50.14
Sibling status				professional title			
	Without sibling	115	31.68		junior RN	287	79.06
	With sibling	248	68.32		middle RN	59	16.25
Marriage					senior RN	17	4.68
	Married	216	59.50	job title			
	Unmarried	147	40.50		nurse	341	93.94
Children status					head nurse	22	6.06
	Have a child	187	51.52	employment type			
	Not have a child	176	48.48		permanent	84	23.14
Educational level					personnel agency	98	27.00
	Secondary	2	0.55		contract	181	49.86
	Diploma	44	12.12	location			
	Bachelor	316	87.05		countryside	88	24.24
	Master	1	0.28		city	275	75.76

### 4.2 Comparison of burnout, empathy and coping style according to socio‐demographic data

The mean scores of burnout, empathy, positive coping style and negative coping style were 39.27 (*SD* 11.48), 111.10 (*SD* 12.18), 24.75 (*SD* 5.48) and 8.94 (*SD* 4.11), respectively. Using the cut‐off scores recommended by Kalimo et al. (2003), most of the nurses reported some burnout (*N* = 262, 72.18%), a few nurses had severe burnout (*N* = 22, 6.06%), whereas the remaining nurses (*N* = 79, 21.76%) had no burnout.

We performed the comparison of burnout, empathy and coping style among different socio‐demographic subgroups using independent *t* test or ANOVA (Table 2). For burnout, we observed statistically significant differences in different marital conditions(*p* = .017), which suggested that a plausible reason for the higher burnout score reported by married nurses (32.20 *SD* 11.87) compared with unmarried nurses (30.17 *SD* 11.61) could be influenced by the conditions and responsibilities of married life. Professional level, job title and employment type were influence factors to the level of empathy in nurses. Head nurses (116.55 *SD* 13.06) had higher empathy scores compared with staff nurses (110.74 Sd 12.06), whereas senior RNs (117.82 *SD* 14.52) had higher scores compared with junior RNs (110.24 Sd 12.15). Further, “personnel agency” nurses (i.e. one type of non‐permanent nurses) (107.86 Sd 12.43) had lower empathy scores compared with permanent (113.21 *SD* 21.36) and contract nurses (11.87 Sd 11.67). Senior RNs (28.18 *SD* 5.21) tended to use a positive coping style more frequently compared with mid‐level (25.32 *SD* 5.84) and junior RNs (24.45 *SD* 5.36). As for negative coping, there were no significant differences observed among different socio‐demographic subgroups.

**Table 2:** Comparison of burnout, empathy ability and coping style in different social‐demographic subgroups

Variable	Category	Burnout					Empathy ability					Positive coping style					Negative coping style				
		Mean	*SD*	*t*	*F*	*p*	Mean	*SD*	*t*	*F*	*p*	Mean	*SD*	*t*	*F*	*p*	Mean	*SD*	*t*	*F*	*p*
Gender				0.519		.604			−0.638		.524			1.775		.184			0.530		.467
	Male	34.13	7.66				108.38	12.45				25.38	3.62				9.13	4.85			
	Female	31.92	11.92				111.16	12.19				24.75	5.52				8.96	4.08			
Sibling				1.683		.093			0.203		.84			0.136		.712			1.834		.177
	Without sibling	33.50	10.75				111.29	12.85				24.65	5.60				9.33	4.27			
	With sibling	31.26	12.27				111.01	11.88				24.82	5.43				8.79	4.00			
Marriage				2.408		.017*			−0.051		.959			0.165		.684			2.101		.148
	Married	33.20	11.87				111.07	11.97				24.44	5.57				9.20	3.91			
	Unmarried	30.17	11.61				111.14	12.52				25.26	5.32				8.62	4.34			
Children status				1.575		.116			0.336		.737			2.042		.154			2.558		.111
	Have children	32.92	11.66				111.30	12.18				24.29	5.75				9.26	3.90			
	Not have a child	30.97	11.98				110.88	12.22				25.27	5.14				8.65	4.27			
Educational level					0.098	.961				0.235	.872				0.599	.616				0.705	.550
	Secondary	34.50	14.85				104.50	0.71				20.00	2.83				11.50	0.71			
	Diploma	31.45	11.06				111.66	13.06				24.39	6.24				8.59	4.33			
	Bachelor	32.02	11.98				111.05	12.12				24.85	5.38				9.01	4.07			
Work unit				1.232		.219			−0.598		.550			0.760		.384			0.553		.458
	Medical unit	32.74	13.03				110.71	13.04				24.35	5.22				8.87	4.21			
	Surgical unit	31.21	10.51				111.48	11.28				25.19	5.71				9.06	3.97			
Professional level					2.163	.116				4.387	.013*				4.142	.017*				1.875	.155
	Junior RN	32.16	11.62				110.24	12.15				24.45	5.36				8.87	4.10			
	Middle RN	33.58	10.93				113.34	10.85				25.32	5.84				9.76	3.72			
	Senior RN	26.94	13.29				117.82	14.51				28.18	5.21				7.82	4.94			
Job title				1.236		.217			−2.176		.030*			0.001		.982			0.274		.601
	Nurse	32.34	11.59				110.74	12.06				24.56	5.44				8.99	4.06			
	Head nurse	29.18	12.16				116.55	13.06				27.95	5.24				8.64	4.64			
Employment type					1.856	.158				5.216	.006**				0.740	.478				0.054	.948
	Permanent	31.58	11.07				113.21	12.36				24.62	5.51				9.08	4.28			
	Personnel agency	34.07	11.38				107.86	12.43				24.29	5.90				8.97	3.71			
	Contract	31.36	11.96				111.87	11.67				25.10	5.23				8.91	4.22			
Location				−1.432		.153			−0.246		.806			1.164		.281			3.643		.057
	Country	37.75	11.58				110.82	11.78				25.10	4.86				8.37	3.62			
	City	39.76	11.42				111.19	12.33				24.66	5.66				9.15	4.22			

* p<0.05; ^**^p<0.01.

### 4.3 Correlation among burnout, coping strategy and empathy

Pearson correlation analysis was performed to test the correlation among the subscales of burnout, coping strategy and empathy, and the results are presented in Table 3. With exception of emotional exhaustion and emotional experience, emotional exhaustion and personal accomplishment, as well as negative coping and personal accomplishment, the correlations among the variables were significant (*p* < .05). Positive coping style had significantly negative correlations with three subscales of burnout (*p* < .01), whereas negative copying style had significantly positive correlations with the subscales of emotional exhaustions (*p* < .01) and depersonalization (*p* < .01). In addition, the subscales of empathy showed significantly negative correlations with three subscales of burnout (*p* < .01) with exception of emotional experience and emotional.

**Table 3:** Correlation among burnout, coping strategy and empathy ability according to Pearson correlation

Variables	1	2	3	4	5	6	7	8
1. Emotional exhaustion	1.000							
2. Depersonalization	0.740**	1.000						
3. Personal accomplishment	−0.068	0.104*	1.000					
4. Positive coping	−0.264**	−0.206**	−0.235**	1.000				
5. Negative coping	0.249**	0.304**	0.092	0.069	1.000			
6. psychological cognition	−0.213**	−0.278**	−0.318**	0.341**	−0.275**	1.000		
7. Emotional experience	−0.047	−0.193**	−0.302**	0.245**	−0.131*	0.596**	1.000	
8. Helping behaviour	−0.143**	−0.234**	−0.369**	0.334**	−0.230**	0.744**	0.563**	1.000

* *p* < .05, **p < .01

### 4.4 Testing the proposed model

**Figure 2:**
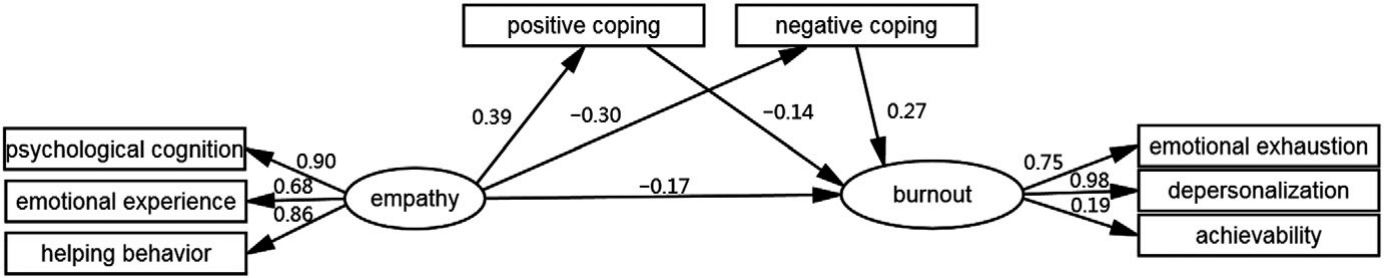
Final tested mode based on structure equation modelling

Maximum‐likelihood estimation was used to test the proposed model. After the inclusion of five correlated errors to improve the model fit, the final model fit was proven to be good (Figure 2). The following values were reve

aled in the model: *X* ^2^ = 18.534, *df* =12, *p* = .100, standardized root mean square residual (SRMR) was 0.025, goodness‐of‐fit index (GFI) was 0.988, adjusted goodness‐of‐fit index (AGFI) was 0.963, normal fit index (NFI) was 0.983, incremental fit index (IFI) was 0.994, and root mean square error of approximation (RMSEA) was 0.039 (Figure 2). Empathy was positively associated with positive coping (standard error [SE] =0.061) (standardized coefficient [β] = 0.38,* p* < .01) and negatively associated with negative coping (SE = 0.071, β = −0.30). Positive coping was negatively associated with burnout (SE = 0.036, β = −0.14), whereas negative coping was positively associated with burnout (SE = 0.048, β = 0.27). In addition, empathy was negatively associated with burnout (SE = 0.056, β = −0.17). Positive and negative coping partially mediated the correlation between empathy and burnout. The mediating effect accounted for 44.6% of the total effect.

## 5 DISCUSSION

According to the results, the mean score for personal accomplishment was higher compared with emotional exhaustion and depersonalization. This difference demonstrated that personal accomplishment plays a critical role in job burnout in medical and surgical wards, which was highlighted by Wang, Liu and Wang (2015): they found that decreased personal accomplishment is strongly correlated with nurses’ job burnout. This outcome meant that the nurses were not satisfied with their actual work results and had confused and negative attitudes towards the meaning of their work. The repetitive nature of daily nursing work makes it more difficult for nurses to achieve satisfaction and improve their sense of achievement in nursing positions. Additionally, patients in China usually trust physicians more than nurses, which has also contributed to the lowered sense of accomplishment experienced by nurses (Cao, Lu, & Liu, 2010). Meanwhile, about four‐fifths of the nurses in the study reported burnout, with 6.06% reporting high burnout. The reported burnout from this study was lower than that of another study conducted in Shanghai (Lu, Ruan, Xing, & Hu, 2015), which has a greater shortage of nurses, a higher social burden on nurses and a faster pace of life.

Our results suggested that married nurses experienced higher job burnout than their unmarried peers. Nurses who are married and have better job experience become the core support in their workplace; they are constantly required to guide and support nursing students and less experienced nurses (Chao, Shih, & Hsu, 2016). As such, they work with more burden and undertake more responsibilities and workload compared with younger and single nurses. Role conflict between work and family has been found to be positively related to burnout (Garcia‐Izquierdo & Rios‐Risquez, 2012). Moreover, married nurses perform daily nursing work with high proficiency and without any difficulty because of their rich clinical experience. Unchallenging work saps employees’ motivation, which causes and low personal accomplishment (Yin, 2015).

The results showed that the mean score of the participants’ level of empathy was slightly higher than the median value, which indicated that the nurses’ level of empathy was between the middle and higher ranges. This finding could be related to the development of High Quality Nursing Service, implemented nationally since 2011 in China. The purpose of this programme was to place patients at the centre of care and strengthen the nurses’ skills in basic nursing care (Meng, Li, & Liu, 2018). Therefore, nurses pay more attention to the psychological status of patients and they become more sensitive towards patients’ feelings.

Our results demonstrated that both senior RNs and head nurses scored higher in empathy compared with junior RNs and staff nurses. Senior RNs usually have more than ten years of work experience in nursing and the greater work and life experience amassed by senior nurses compared with their junior colleagues give them advanced practical and theoretical knowledge (Humpel & Caputi, 2001). Thus, this advantage enables them to be more sensitive to their patients’ behaviour and emotions, leading them to act altruistically, such as meeting and assisting their patients’ needs promptly (Humpel & Caputi, 2001). Moreover, head nurses have received more opportunities for psychological training than staff nurses and this places them at an advantage to develop and practice empathy towards patients and their peers through their work. In addition, head nurses, as managers of and role models to the staff nurses, would pay particular attention to their own behaviours (Kalimo et al., 2003) to set an example to the nursing team in demonstrating empathy to patients.

Personnel agency nurses reported the lowest empathy score of 107.86 (*SD* 12.43), compared with permanent and contract nurses. Different employment types result in different salary and welfare benefits (Zhao, 2020). Permanent nurses receive the highest salary and welfare benefits, followed by personnel agency and contract nurses, which are off‐staff nurses. After the reform in the personnel system in China, advocated in the health sector since 2002 (Wang, 2019), there is a growing demand for personnel agency and contract nurses, whereas the number of nurses with permanent employment type has reduced and even disappeared in some hospitals. In other words, permanent nurses are normally older than personnel agency and contract nurses. In this study, the average work years of permanent nurses was 16.51, higher than that of personnel agency (6.59 years) and contract nurses (4.18 years). Nurses who are older have more life experience and could accurately identify patients’ needs and empathize with them. Moreover, although personnel agency and contract nurses have the same job responsibilities as permanent nurses, they are paid a lower salary and receive fewer welfare or promotion opportunities compared with permanent nurses (Wang, 2019). This discrepancy might explain the higher level of empathy of permanent nurses. Personnel agency nurses often have a higher academic background and stronger professional abilities than contract nurses. Although an increasing number of nursing educators recognize the importance of psychological well‐being, medical knowledge and skills continue to play a vital part in the nursing curriculum. Thus, personnel agency nurses pay more attention to their professional knowledge instead of patients’ psychological status, which translates to a lowered level of empathy. Meanwhile, the professional ability of contract nurses is relatively weak. Contract nurses also tend to focus more on patients’ physical conditions compared with personnel agency nurses, which could be the reason for contract nurses’ higher level of empathy than personnel agency nurses. To improve the working enthusiasm and efficiency of employees, as well as avoid agency failure and other negative phenomena, many hospitals have begun implementing a flexible management system, where different employment types can be adjusted according to the employee's performance (Gai, 2019).

The nurses’ positive coping style score exceeded the national average, whereas their negative coping style score was lower compared with the national average. The results were consistent with the findings of Shi (2013), which found that most nurses obtain good adjustment ability and resistance to strong pressure. The result of nurses’ coping style score might closely relate to the relatively low stress in nurses’ working units, high educational level of the nurses, extensive network of contacts and strong communication ability. Our results also showed that senior RNs had higher scores than mid‐level and junior RNs in the area of positive coping style. This finding suggests that senior nurses are more inclined to adopt positive coping methods, which may be owing to their extensive work experience, strong job autonomy, good career development prospects and complete social support system. All these factors enable them to deal with their stress at work.

The results revealed the positive correlation between negative coping style and burnout and the negative correlations between positive coping style and burnout and between the level of empathy and burnout. The effects of positive and negative coping strategies on job burnout were consistent with prior findings (Li & Li, 2012; Xie et al., 2013). Job burnout is an extreme reaction when an individual cannot cope with stress smoothly. It is a condition of emotional exhaustion, and it is an attitude and behaviour under long‐term stress. Prolonged high job stress and inappropriate coping strategies can lead to job burnout. Positive coping styles can help people solve problems and reduce the lack of fulfilment at work. In contrast, negative coping styles cannot solve problems but rather lead to worse emotional conditions and cause people to be indifferent and unsympathetic towards others (Xie, Zhang, & Lin, 2005).

High levels of empathy tended to reduce job burnout and vice versa, in line with the findings of Qi, Hou, Gu, and Chang (2011). Hoffman (2001) emphasized that empathy is the ability to understand others’ thoughts, situation and feelings from their perspective. People with high levels of empathy can be aware of the needs and emotions of others more deeply. In addition, empathy can help individuals be less self‐centred and thus learn to understand, care for, tolerate and recognize others’ needs. In nursing practice, nurses with high levels of empathy can establish good nurse–patient relationships easily and thus obtain higher job satisfaction (Song, 2015). Moreover, our results suggested that coping style may be an important intervening factor between empathy and burnout. Both positive coping and negative coping were mediators in the correlation between empathy and burnout. Individuals can deal with difficulties by mobilizing different coping styles, which eventually produces different levels of burnout. Nurses with strong empathy are more positive and less negative in responding to stress (Jia，^2018^). Indeed, individuals with a strong ability to empathize have a stronger perception of social support and psychological resilience (Pang, Zhao, & Su, 2019). As empathy is a skill in interpersonal communication, nurses with strong empathy often have good interpersonal relationships and strong social support systems. Thus, when they encounter stressful events, they are more inclined to adjust their mentality and actively seek help from others to respond accordingly. Meanwhile, nurses with poor empathy skills have poor psychological resilience, poor ability to perceive external support and often choose to respond negatively to stressful events.

Ding et al. (2015) found that nurses with positive coping skills experience lower levels of emotional exhaustion, depersonalization and personal accomplishment. Positive coping styles help nurses gradually leave their negative predicament. Psychological adjustment, conversation, experience and other solutions can help nurses alleviate stress, calmly deal with problems, maintain a good psychological state and carry a high sense of personal accomplishment. However, using negative coping styles to deal with occupational problems will lead to the continuous accumulation of problems, which cannot be effectively resolved and personal abilities will not be improved. Further, continuing to adopt negative coping styles will lead to constant self‐denial, lack of personal accomplishment and psychological problems, such as emotional exhaustion and depersonalization (Xie et al., 2013).

### 5.1 Limitations

This study has a few limitations. First, we used a cross‐sectional design, with data being collected at only one point in time. Therefore, the study could not identify the changes and trends over time and as such might provide weak evidence of correlation among burnout, coping strategies and empathy. Second, data were collected only in general surgical and medical wards; thus, the convenience sample may not be representative of the targeted population and the results may be biased. Future studies should be conducted in more areas, such as the emergency department and intensive care unit, to ensure a representative and diverse sample that could more accurately reflect the nursing population. Nonetheless, the effect of the limitations might be diminished by the diverse dimensions included during measurement.

## 6 CONCLUSION

Nurse burnout was prevalent among nurses in China, and it was associated with coping strategies and empathy. The present findings suggested that enhancing empathy skills and positive coping strategy, and the reduction of negative coping strategy, may lead to a reduction of burnout in nurses. To reduce work‐related burnout, hospital administrators, nurse managers and policymakers should provide continued training, education and mentoring for nurses. Moreover, they need to take action to improve nurses’ perception of being supported not only in their work but also by society at large. These measures may help achieve the goal of increasing job satisfaction for nurses and the quality of patient care provided.

## CONFLICT OF INTEREST

The authors declare that they have no competing interests.
